# Revealing gene expression links between milk fat globules and mammary glands in rodents via transcriptomics

**DOI:** 10.3389/fvets.2025.1555705

**Published:** 2025-05-13

**Authors:** Hancai Jiang, Xiaoxian Xu, Xinhui Song, Qingyou Liu, Kuiqing Cui, Hui Li, Jieping Huang, Zhipeng Li

**Affiliations:** ^1^State Key Laboratory for Conservation and Utilization of Subtropical Agro-Bioresources, College of Animal Science and Technology, Guangxi University, Nanning, China; ^2^Guangdong Provincial Key Laboratory of Animal Molecular Design and Precise Breeding, School of Life Science and Engineering, Foshan University, Foshan, China

**Keywords:** mammary gland, milk fat globules, lactation traits, gene expression profiles, whole-transcriptome sequencing

## Abstract

Currently, most studies on lactation-related traits and gene expression rely on invasive techniques to obtain mammary tissue. These methods are not only difficult to perform but also limit the availability of samples. Therefore, this study aimed to utilize whole transcriptome sequencing to investigate the gene expression profiles of Golden hamsters (Gh, *n* = 5) and Kunming mice (Km, *n* = 5). It compared the transcriptome expression between milk fat globules (MFG) and the mammary gland (MG), identified candidate genes and pathways associated with lactation traits, and assessed the potential of MFG as an effective alternative to MG. The data showed that a total of 21,360 genes were identified in the Gh group, with 66.5% of the mRNAs showing no differential expression between MG and MFG. In the Km group, a total of 44,248 genes were identified, with non-differentially expressed genes (NDEGs) accounting for 58.8%. Additionally, the majority of ncRNA data consisted of NDEGs. In both groups, approximately 80% of miRNA data were NDEGs. Notably, the proportion of NDEGs in circRNA data approached 100%. Enrichment analysis revealed that NDEGs from both groups were significantly enriched in several pathways, including the MAPK signaling pathway, PI3K-Akt signaling pathway, JAK-STAT signaling pathway, and prolactin signaling pathway, all of which are closely associated with lactation traits and the lactation process. Furthermore, we identified various ncRNAs that regulate the expression of target genes either directly or indirectly, thereby influencing the lactation process. This study validates MFG as a reliable substitute for MG, with potential applications in improving dairy science. By identifying key genes and pathways, it provides new insights for optimizing genetic selection and breeding strategies. It also supports the improvement of dairy animal management practices.

## 1 Introduction

Common dairy livestock, including Holstein cattle, buffaloes, goats, camels, and others, are primarily valued for their milk production as a key economic trait. High-quality milk sources are characterized by a comprehensive nutritional profile, offering abundant nutrients essential for the growth and development of both animal offspring and humans (1). Investigating the lactation traits of dairy livestock is critical for improving milk production performance, contributing to the supply of higher-quality milk products with more comprehensive nutritional value. Milk production and secretion represent a complex physiological process, wherein various nutrients are absorbed and utilized to synthesize milk proteins, milk fat, and lactose. This process is mainly regulated by gene expression and post-transcriptional modification. Furthermore, non-coding RNA also plays an important regulatory role in milk production and secretion (2–4). Identification and analysis of candidate genes related to milk production traits in dairy livestock have important guiding significance for improving milk production performance, developing genetic potential and molecular breeding (5–7).

Mammary gland (MG) serves as the site of milk secretion and a primary material for studying lactation traits. Therefore, analyzing molecular functions in MG can offer valuable insights into the regulatory mechanisms of lactation and provide a foundation for molecular breeding of high-quality dairy animals. However, traditional MG collection methods are largely impractical for large, high-quality lactating livestock. This is due to their significant economic value and the high costs associated with testing. Secondly, traditional udder collection methods require *in vivo* sampling of MG, which is invasive and often involves damaging tissue. This approach carries the risk of reducing dairy animals’ production performance, an increased likelihood of udder inflammation, and the potential limitation that local tissue samples may not accurately represent the entire MG (8). The limited availability of MG materials has notably hindered the advancement of studies on animal lactation traits. During lactation, mammary epithelial cells (MECs) secrete fat into milk through apical secretion, forming cytoplasmic lipid droplets that are encapsulated by the plasma membrane to create milk fat globules (MFG) (9–11). MFG is abundant in milk, existing as small droplets. During its formation, a portion of the cytoplasm remains in the outermost layer of the fat droplets and membrane, often forming a crescent-like structure. This crescent contains various proteins, including domains of cytoplasmic-integrated membrane proteins and membrane-associated peripheral proteins from MECs (12–14). The unique structure of MFG, combined with its easy availability, makes it an ideal substitute for MG. The study by Maningat et al. (15) demonstrated that MFG RNA serves as a non-invasive alternative to MG RNA for studying lactation-related genes in humans. Brenaut et al. (16) further validated the reliability of MFG RNA in investigating bovine lactation processes and immune responses. Cánovas et al. (17) compared five RNA sources and confirmed MFG RNA as a suitable substitute for MG RNA in lactating dairy cows. In summary, MFG RNA is an effective, non-invasive tool for lactation research, with gene expression patterns similar to MG. This approach overcomes traditional sampling limitations, preserving animal productivity and reducing costs. However, there is limited literature available on using MFG as a replacement for MG, and further development of technical methods and related studies is needed to enhance its representativeness.

Currently, most research on lactation related traits and gene expression relies on invasive techniques, such as tissue biopsy or slaughter, to analyze gene expression during lactation. Not only are these methods difficult to perform, but they also limit the availability of samples. In contrast, MFG is a novel non-invasive method, particularly valuable for species where sampling is challenging or limited. Therefore, the aim of this study was to analyze the gene expression profiles of MG and MFG through whole transcriptome sequencing, identify key candidate genes and associated pathways, and evaluate the feasibility of using MFG as a non-invasive alternative to MG in rodents (Golden hamsters and Kunming mice). Notably, this study uses hamsters and mice as experimental animals, as they are widely used in laboratory settings and can provide insights into general lactation patterns that may be applicable to other dairy animals. Additionally, the collection of MG samples from large dairy animals presents considerable challenges and costs. The study comprehensively examined both differential and non-differential aspects of gene expression profiles to provide a deeper understanding of lactation traits research. Furthermore, this study provides a valuable foundation for non-invasive research in large mammalian species, with potential implications for improving dairy animal breeding strategies and advancing livestock management practices.

## 2 Materials and methods

### 2.1 Sample collection

The animals in this experiment have received the humanitarian care outlined in the National Institutes of Health “Laboratory Animal Care and Use Guidelines.” Animal experiments were approved by the Ethical Review Board of Animal Experiments of Guangxi University (Approval No. GXU-2022-192). Golden hamsters (Gh, *n* = 5) and Kunming mice (Km, *n* = 5) were raised to the peak lactation period with free access to food and water. Female mice were selected based on having 2 to 3 litters and being in the lactation period of 6 to 10 days. At this stage, the lactation ability of mice was the strongest and stable. Each group consists of five independent biological replicates. Milk samples were collected first, followed by MG from the same animals. To promote milk accumulation, the mother rats were separated from their pups the day before collection. Under anesthesia, an Automated Milker for Rat and Mouse (Muromachi, Tokyo, Japan) was used. 1.0 to 1.5 mL of milk was collected from each Gh and Km. RNA was extracted from samples within 1 h after collection using the Trizol method. NanoDrop 2000 (Thermo, Waltham, United States) and agarose gel electrophoresis were used to detect RNA concentration and integrity. RNA samples exhibiting three intact bands and an RNA integrity number (RIN) value greater than 7 were selected for library construction and sequencing. The MFG and MG of Gh group were named G-MFG and G-MG, respectively. The MFG and MG of Km group were named K-MFG and K-MG, respectively.

### 2.2 Library construction and sequencing

Library construction and sequencing were performed by BGI Genomics (Shenzhen, China). In this study, two sequencing libraries were constructed: an rRNA-depleted, strand-specific library containing lncRNA, circRNA, and mRNA, and a miRNA library. After library construction, the concentration and quality of the libraries were assessed. The concentration was measured using a Qubit fluorometer (Thermo, Waltham, United States), while the size distribution and integrity of the RNA fragments were evaluated with a Bioanalyzer (Agilent Technologies, Santa Clara, United States). Only libraries that met the required concentration and quality standards were selected for sequencing. The strand-specific library sequencing platform uses DNBSEQ and runs the PE100 double-end sequencing program for sequencing. The miRNA library sequencing platform uses DNBSEQ and runs the SE50 program for sequencing. The original sequencing data were stored in the NCBI sequence reading file (Accession No. PRJNA1003892), and located in the hamster (*Cricetulus griseus*) genome CriGri_1.0 and the mouse (*Mus musculus*) genome GRCm39.

### 2.3 Analysis of mRNA biological information

Fastp software (v0.19.4) was used to filter the raw data obtained by sequencing and obtain high-quality sequences (Clean Data) after GC content processing. The Clean Data were compared to the reference genome using Hisat2 software (v22.2.1), and then the reads were spliced into transcripts for quantification using StringTie software (v2.1.5) based on the results of the comparison to the genome. By comparing with the existing genome annotation information, identify previously unannotated transcription regions and discover new transcripts and new genes in Gh and Km. Gene expression levels were quantified using the fragments per kilobase of transcript per million mapped reads (FPKM) method. Differential expression analysis was performed based on these FPKM values. To control for false discovery due to multiple testing, the *p*-values were adjusted using the Benjamini–Hochberg procedure. Genes with FDR <0.05 and |log_2_(fold change)| ≥1 were considered as significantly differentially expressed between groups. To gain further insights into the functions and pathways of non-differentially expressed genes (NDEGs) and differentially expressed genes (DEGs) across groups, Gene Ontology (GO) and Kyoto Encyclopedia of Genes and Genomes (KEGG) pathway enrichment analyses were conducted using ClusterProfiler software (v3.18). The protein–protein interaction (PPI) network was constructed using the STRING database (v12.0), with a confidence score threshold set to >0.7. The network was visualized and analyzed using Cytoscape software (v3.8.2). The Network Analyzer tool was used to identify the proteins with the highest connectivity, and the corresponding genes were considered to play a central role in lactation-related pathways.

### 2.4 Identification of ncRNA and prediction of its target genes

LncRNA transcripts with a class code of “i” (intergenic), “x” (antisense to known coding genes), “u” (unknown function), “o” (overlapping with a coding gene), or “e” (enhancer-associated) and an FPKM value ≥0.1 were selected. Transcripts shorter than 200 bp or containing fewer than two exons were excluded. The selected candidate lncRNAs were then analyzed using CPC, CNCI, CPAT, and Pfam protein domain prediction tools. The expression abundance of lncRNA transcripts after filtering was quantified using the FPKM method. To control for multiple testing, *p*-values were adjusted using the Benjamini–Hochberg procedure. Differential expression analysis was performed with thresholds of FDR <0.05 and |log_2_(fold change)| ≥1. Perl script was utilized to identify the adjacent genes within a 100 kb range upstream and downstream of lncRNAs as their cis-target genes.

The analysis of cicrRNA mainly involves the following steps. Sequencing quality was assessed using FastQC software, and high-throughput sequencing reads were processed with fastp software. circRNAs were then identified, quantified, and filtered using CIRIquant software (v2.0.6), retaining transcripts with expression levels ≥2 for further analysis. The raw counts were normalized using the transcripts per million (TPM) method to account for sequencing depth and gene length. The differential expression analysis was performed using edgeR software (v4.0.16), with *p*-values adjusted for multiple testing using the Benjamini–Hochberg procedure. The thresholds were set at FDR <0.05 and |log_2_(fold change)| ≥1. Finally, the prediction of circRNA target genes was also conducted using CIRIquant software.

The miRNA sequences were aligned against the known miRNA sequences in the miRBase database (v22.1), permitting a maximum of one mismatch. The alignment encompassed two nucleotides upstream and five nucleotides downstream of each sequence. Reads that met these alignment criteria were classified as known miRNAs. The expression levels were quantified using the TPM algorithm, and differential expression analysis was performed using DESeq2. *p*-values were adjusted for multiple testing using the Benjamini–Hochberg procedure, with thresholds set at FDR <0.05 and |log_2_(fold change)| ≥1. miRNA target genes were predicted using TargetScan and miRDB. ClusterProfiler software (v3.18) was used for GO functional annotation and KEGG pathway enrichment analysis of both non-differential and differential target genes associated with lncRNA, circRNA, and miRNA.

### 2.5 Quantitative reverse transcription polymerase chain reaction (qRT-PCR) analysis

The RNA of MG and MFG was reverse transcribed into cDNA using a RT-PCR kit (Vazyme, Nanjing, China). In order to determine the accuracy of the data, NDEGs (Supplementary Table S1) were randomly selected from mRNA. The primers were designed by Primer Premier 5 software and synthesized by Sangon Biotech Co., Ltd. (Shanghai, China). The SYBR qPCR Master Mix (Vazyme, Shanghai, China) kit was used for q-PCR, and the reaction system and reaction process were referred to the kit instructions. With MG as the control group and glyceraldehyde-3-phosphate dehydrogenase (GAPDH) as the internal reference gene, q-PCR was performed by LightCycler® 96 instrument (Roche, Basel, Switzerland), and the relative expression of the gene was calculated using 2^−ΔΔCT^.

### 2.6 Statistical analysis

The experiment was repeated three times in each group. SPSS (v26.0) software was used for statistical analysis. The values were expressed as mean ± SEM. The statistical differences between the two groups were analyzed by *t*-test and Wilcoxon signed-rank test. *p* < 0.05 was significant, *p* < 0.01 was extremely significant, and *p* > 0.05 indicated no significant difference. The chart is drawn using GraphPad Prism (v 9.0).

## 3 Results

### 3.1 Basic information of RNA sequencing data

#### 3.1.1 Gene expression levels and identification in MG and MFG samples

**Figure 1:**
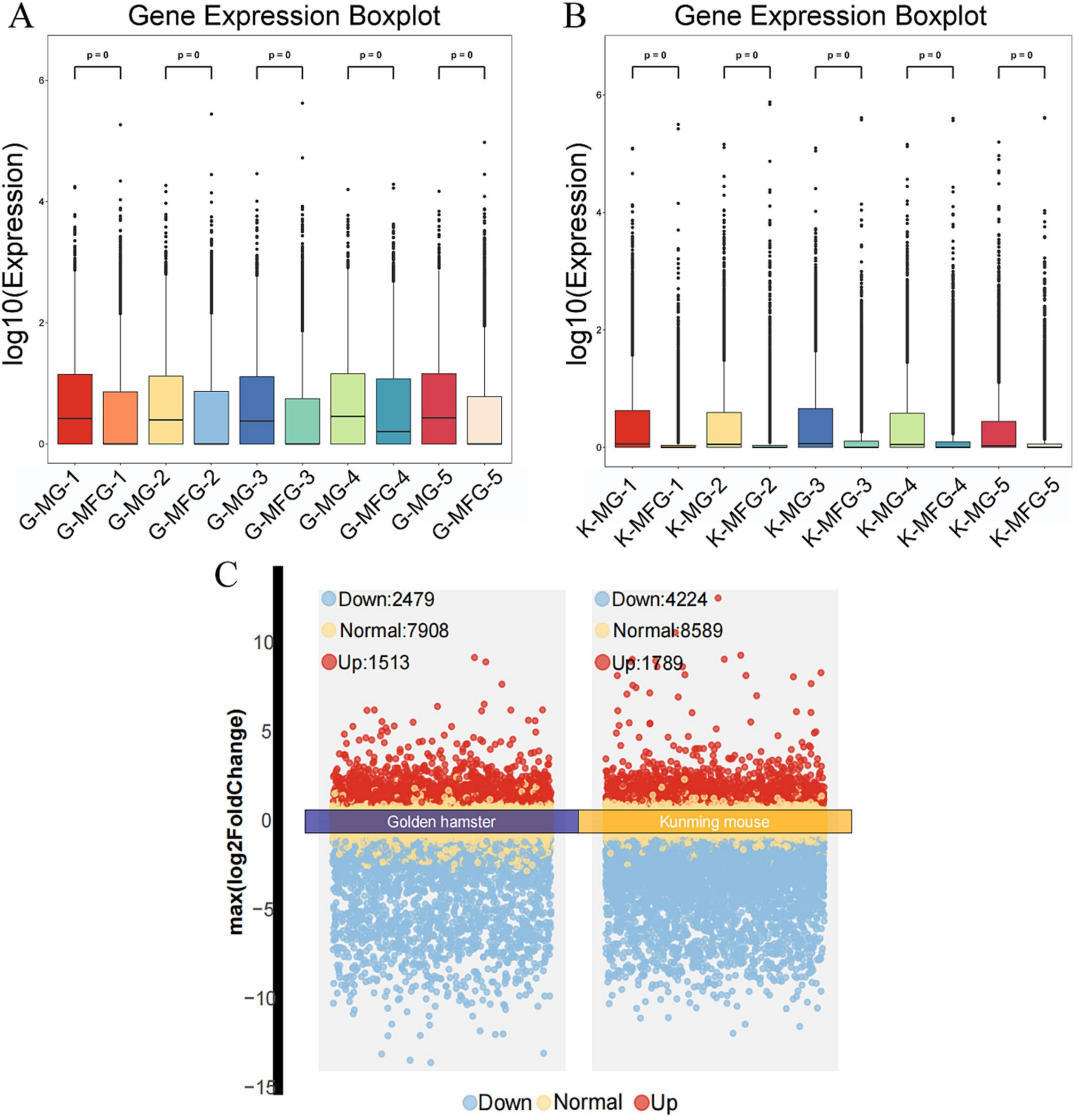
Basic information of transcriptome data. **(A)** Boxplot of expression level of each sample in Gh group (each point represents the expression level of an individual gene, and the boxes summarize the distribution within each sample). **(B)** Boxplot of expression level of each sample in Km group. **(C)** Volcano plots of Gh group and Km group (FDR <0.05 and |log2 (fold change)| ≥1).

The boxplot results show that the total gene expression levels in MG samples were higher than those in MFG samples for both the Gh and Km groups (Figures 1A,B). In the mRNA data of the Gh group, 21,360 genes were identified, including 318 newly discovered genes, 279 of which were functionally annotated. Specifically, 2,586 genes were uniquely expressed in MG, 166 in MFG, and 12,624 genes (59.1% of the total) were co-expressed in both MG and MFG. Similarly, in the Km group, 44,248 genes were identified, with 909 newly discovered genes, 681 of which were functionally annotated. Of these, 8,191 genes were uniquely expressed in MG, 946 in MFG, and 23,191 (52.4%) were co-expressed in both groups.

#### 3.1.2 Differential gene expression analysis in Gh and Km groups

Gene expression analysis revealed that, compared to MG, the Gh group had 7,908 NDEGs and 3,992 DEGs, with 1,513 upregulated and 2,479 downregulated. This indicates that 66.5% of the genes were non-differentially expressed between MG and MFG. In the Km group, there were 8,589 NDEGs and 6,013 DEGs, including 1,789 upregulated and 4,224 downregulated genes (Figure 1C). NDEGs accounted for 58.8% in this group. In the Gh and Km groups, a large number of DEGs and NDEGs were identified, which are closely associated with lactation traits (Supplementary Tables S2, S3).

### 3.2 Expression of mRNA in Gh group

#### 3.2.1 Functional annotation and pathway enrichment analysis of NDEGs

**Figure 2:**
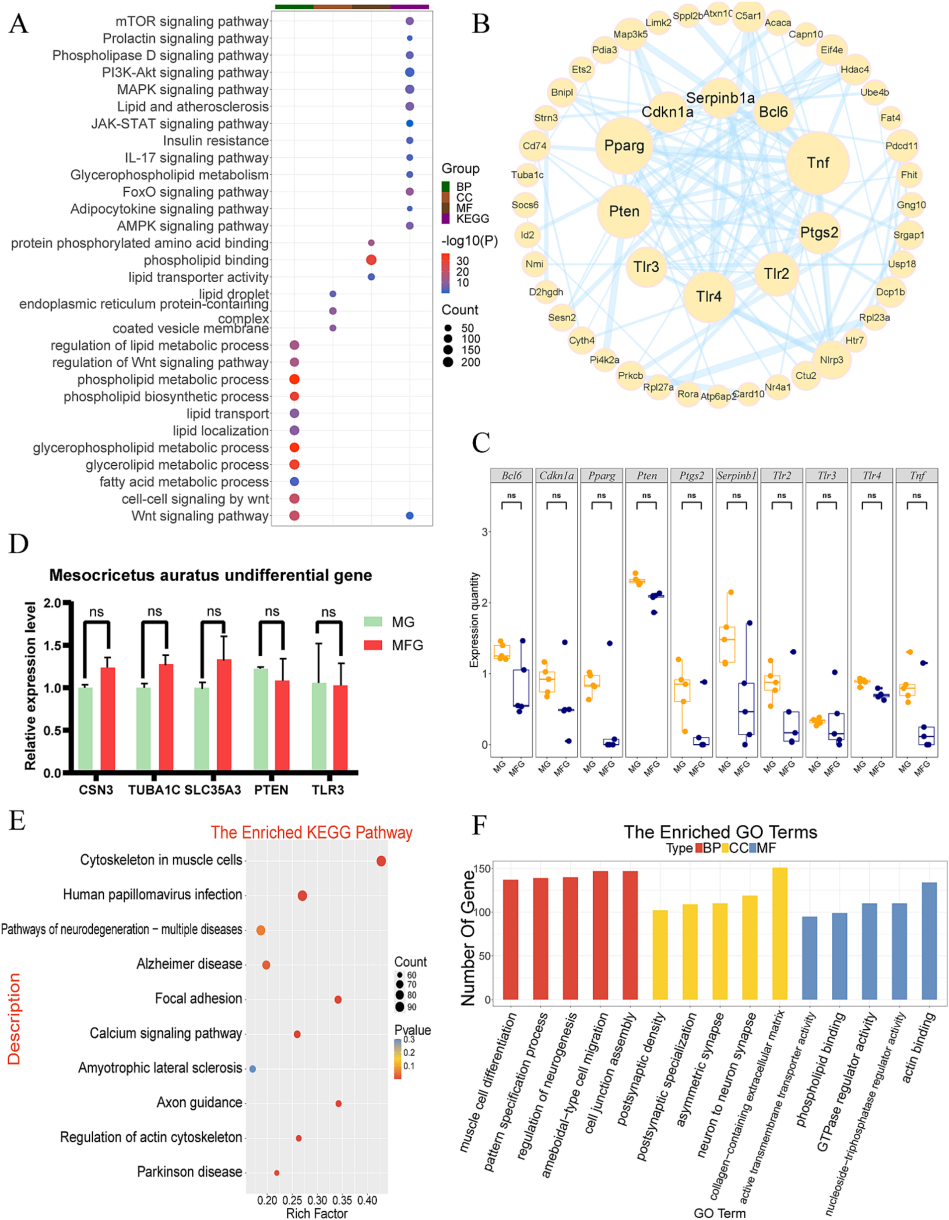
Expression patterns and functional analysis of mRNA in the Gh group. **(A)** KEGG and GO enrichment analysis of Gh NDEGs (each dot represents a pathway, with size correlating to the number of related genes and color intensity indicating the statistical significance). **(B)** The PPI network analysis of the Gh group (the more protein nodes, the larger the circle, the closer the connection with other genes, the thicker the link line) **(C)** Gh group gene expression boxplot in MG and MFG. ns indicates no significant difference (*p* > 0.05). **(D)** q-PCR validation of NDEGs in the Gh group. ns indicates no significant difference (*p* > 0.05). **(E)** KEGG enrichment analysis of DEGs in Gh group. **(F)** GO enrichment analysis of DEGs in the Gh group, categorized into biological processes (BP), cellular components (CC), and molecular functions (MF).

GO functional annotation and KEGG pathway enrichment analysis were conducted on the 7,908 NDEGs identified in the Gh group. KEGG analysis revealed that these NDEGs were primarily enriched in pathways such as the PI3K-Akt signaling pathway, MAPK signaling pathway, lipid and atherosclerosis, JAK-STAT signaling pathway, and prolactin signaling pathway, among others (Figure 2A). GO functional annotation showed enrichment in terms related to the phospholipid metabolic process, Wnt signaling pathway, glycerolipid metabolic process, regulation of lipid metabolic processes, and fatty acid metabolic processes (Figure 2A). According to the enrichment, most of the pathways were found to be related to lactation traits and lactation processes. These pathways were selected for PPI mapping to determine the protein interactions between proteins encoded by the genes in these pathways (Figure 2B). It was found that the proteins encoded by the genes *CDKN1A*, *PPARG*, *PTEN*, *TLR3*, *TLR4*, *TLR2*, *PTGS2*, *TNF*, *SERPINB1*, and *BCL6* were most closely related to other proteins in the interaction network, with the largest number of protein nodes. These proteins are likely to play a central role as hub proteins in the regulatory network of lactation-related genes, potentially influencing key processes in lactation regulation. The expression of the above genes in MG and MFG was observed by boxplot (Figure 2C). The results showed that the expression levels of each gene were similar, especially *PTEN*, *TLR4*, and *TLR3*. However, the expression level of each gene in MFG was still slightly lower than that in MG. Five genes related to lactation traits were randomly selected from NDEGs, and their expression levels were detected by q-PCR. The results indicated that the expression pattern of the selected genes was consistent with the expression pattern in RNA-seq (Figure 2D).

#### 3.2.2 Enrichment analysis of DEGs

Additionally, 33.5% of the genes were differentially expressed between MG and MFG, and enrichment analysis was conducted. The results revealed that DEGs were significantly enriched in pathways such as cytoskeleton organization in muscle cells, human papillomavirus infection, neurodegeneration pathways, actin binding, GTPase regulator activity, and others (Figures 2E,F).

### 3.3 Expression of mRNA in Km group

#### 3.3.1 Functional annotation and pathway enrichment analysis of NDEGs

**Figure 3:**
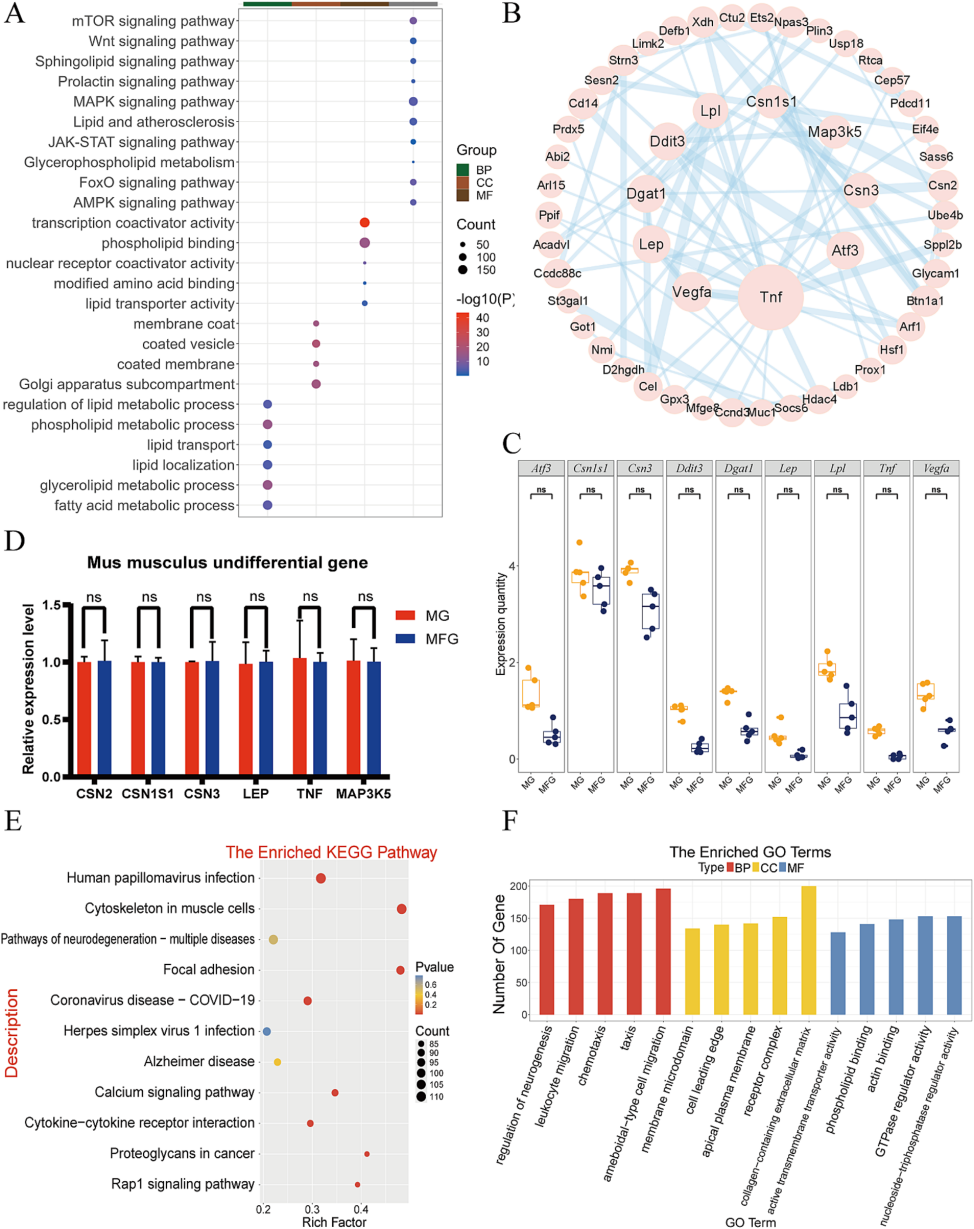
Expression patterns and functional analysis of mRNA in the Km group. **(A)** KEGG and GO enrichment analysis of Km NDEGs. **(B)** The PPI network analysis of the Km group. **(C)** Km group gene expression boxplot in MG and MFG. ns indicates no significant difference (*p* > 0.05). **(D)** q-PCR validation of NDEGs in the Km group. ns indicates no significant difference (*p* > 0.05). **(E)** KEGG enrichment analysis of DEGs in Km group. **(F)** GO enrichment analysis of DEGs in Km group.

GO functional annotation and KEGG pathway enrichment analysis were also performed on 8,589 NDEGs in Km group. KEGG analysis showed that NDEGs were mainly enriched in MAPK signaling pathway, lipid and atherosclerosis, mTOR signaling pathway, JAK-STAT signaling pathway, prolactin signaling pathway, glycerophospholipid metabolism and other pathways (Figure 3A). The GO clustering results showed that the Km group was enrich in related terms such as glycerolipid metabolic, phospholipid metabolic, fatty acid metabolic and lipid localization (Figure 3A). Multiple pathways were also found to be related to lactation traits and lactation processes in the data of Km. These pathways were selected for PPI mapping (Figure 3B). It was found that the proteins encoded by the genes *LPL*, *DDIT3*, *DGAT1*, *LEP*, *VEGFA*, *TNF*, *ATF3*, *CSN3*, and *CSN1S1* were most closely related to other proteins in the interaction network, with the largest number of protein nodes. Boxplot were made for the above genes to observe the expression of each gene in MG and MFG (Figure 3C), and it was found that the expression levels of *CSN1S1*, *LEP*, and *TNF* were particularly similar. Six genes related to lactation traits were randomly selected from NDEGs and their expression levels were detected by q-PCR. The results showed that the expression patterns of the selected genes were consistent with those in RNA-seq (Figure 3D).

#### 3.3.2 Enrichment analysis of DEGs

In addition, 41.2% of the genes were differentially expressed between MG and MFG, and enrichment analysis was performed. The results showed that DEGs were highly enriched in Human papillomavirus infection, cytoskeleton in muscle cells, pathways of neurodegeneration, ameboidal-type cell migration, chemotaxis, taxis, and other pathways (Figures 3E,F).

### 3.4 Identification of lncRNA and prediction of its target genes

#### 3.4.1 Identification and distribution of lncRNAs

**Figure 4:**
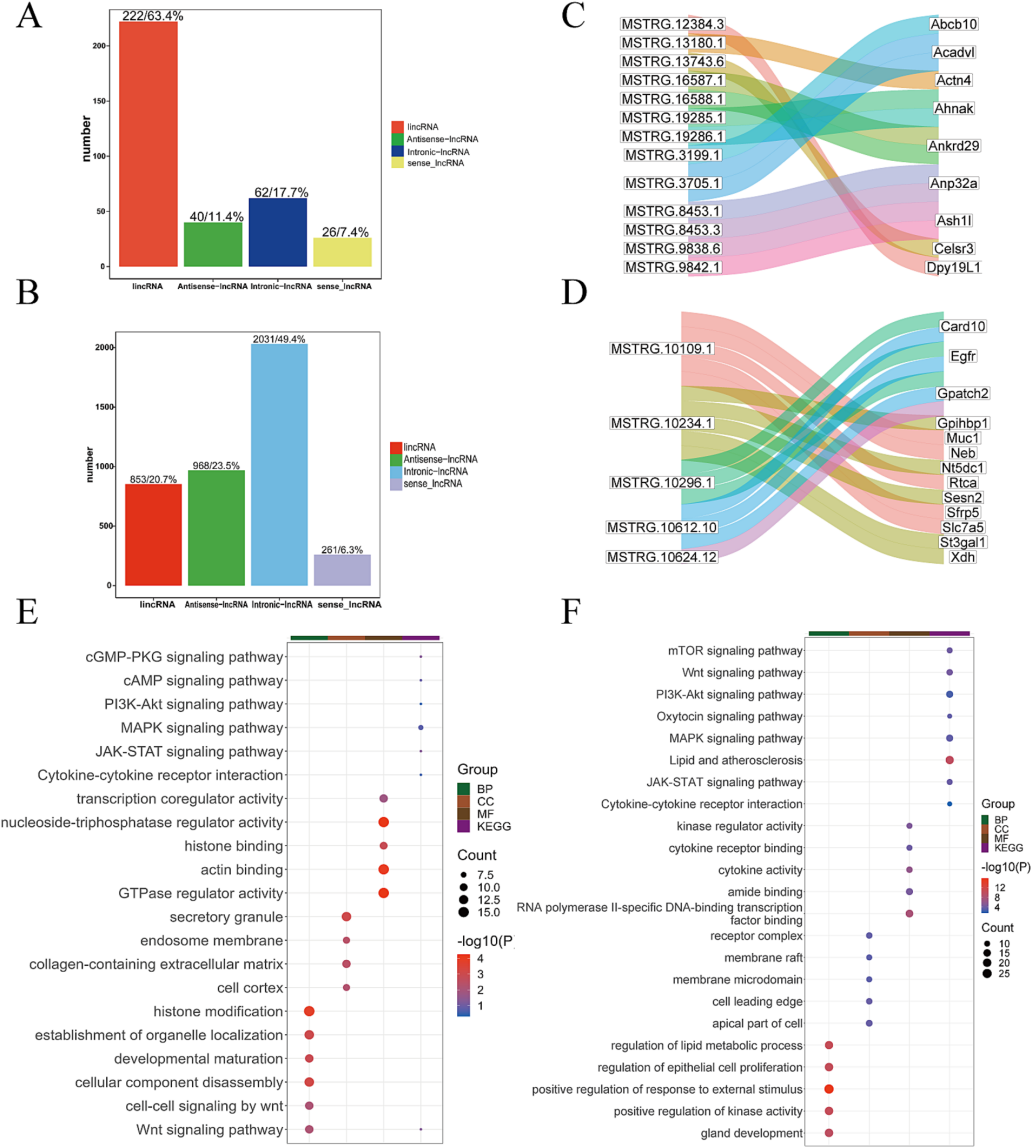
Bioinformatics analysis of lncRNA. **(A)** Statistics of the number of four different types of lncRNAs in the Gh group. **(B)** Statistics of the number of four different types of lncRNAs in the Km group. **(C)** Part of lncRNA transcripts and target gene Sankey diagram in Gh group. **(D)** Part of lncRNA transcripts and target gene Sankey diagram in Km group. **(E)** lncRNA KEGG and GO enrichment analysis in Gh group. **(F)** lncRNA KEGG and GO enrichment analysis in Km group.

Following lncRNA identification and prediction, 350 lncRNA transcripts were identified in the Gh group, while 4,113 lncRNA transcripts were identified in the Km group. The distribution of lincRNAs, antisense ncRNAs, intron-lncRNAs, and sense-lncRNAs in each group is illustrated the figure (Figures 4A,B). Using the criteria of FDR <0.05 and |log_2_(fold change)| ≥1, 157 non-differentially expressed lncRNA transcripts were identified in the Gh group, accounting for 44.9% of the total. These non-differential transcripts were subjected to target gene prediction, resulting in the identification of 404 cis-target genes, including examples such as MSTRG.12384.3 targeting *DPY19L1*, MSTRG.13743.6 targeting *CELSR3*, and MSTRG.3705.1 targeting *ACADVL* (Figure 4C). In the Km group, 959 non-differentially expressed lncRNA transcripts were identified, accounting for 23.3% of the total. These non-differential transcripts were analyzed for target gene prediction, resulting in the identification of 29,180 lncRNA cis-target genes. The non-differential lncRNA transcripts were mapped to target genes, such as MSTRG.10296.1, MSTRG.10612.10 and MSTRG.10109.1, which targeted *GPATCH2*, *EGFR*, and *MUC1*, respectively (Figure 4D).

#### 3.4.2 Functional annotation and pathway enrichment of lncRNA target genes

Further GO functional annotation and KEGG pathway enrichment analysis were performed on the lncRNA target genes of the Gh and Km groups. The most representative GO terms and KEGG pathway results revealed that several pathways were closely associated with lactation, including the PI3K-Akt signaling pathway, oxytocin signaling pathway, MAPK signaling pathway, JAK-STAT signaling pathway, and pathways related to lipid metabolism and atherosclerosis (Figures 4E,F).

### 3.5 Identification, target gene prediction, and functional enrichment analysis of circRNAs

#### 3.5.1 Identification and target gene prediction of circRNAs

**Figure 5:**
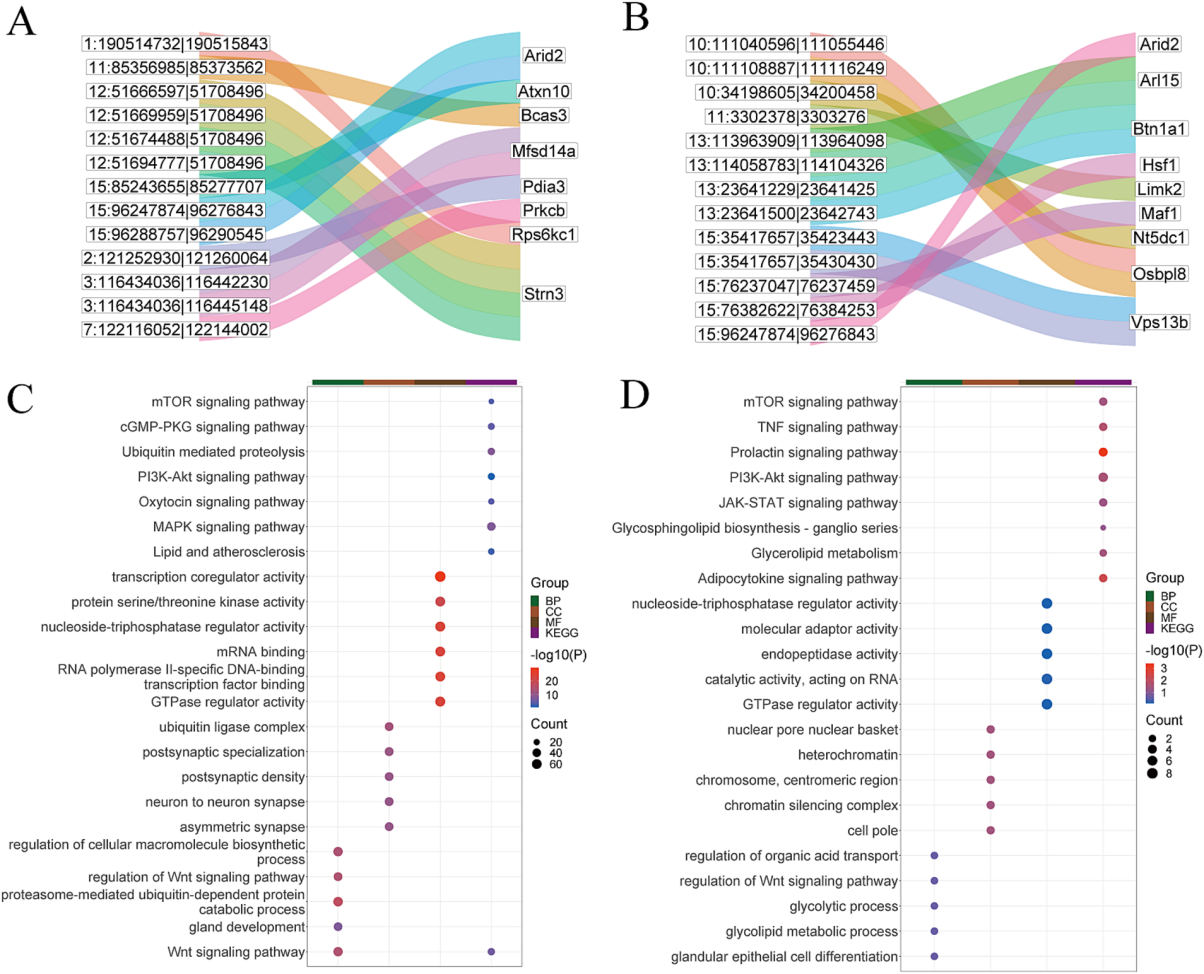
Bioinformatics analysis of circRNA. **(A)** Part of circRNA transcripts and target gene Sankey diagram in Gh group. **(B)** Part of circRNA transcripts and target gene Sankey diagram in Km group. **(C)** KEGG and GO enrichment analysis of circRNAs in Gh group. **(D)** circRNA KEGG and GO enrichment analysis in Km group.

A total of 1,816 circRNA transcripts were identified in the Gh group, with 1,769 being non-differentially expressed, accounting for 97.4% of the total. These non-differential transcripts underwent target gene prediction, resulting in the identification of 1,063 target genes. For example, Chr11: 85356985|85373562, Chr12: 51666597|51708496, Chr15: 85243655|85277707, Chr15: 96247874|96276843 targeted *BCAS3*, *STRN3*, *ATXN10*, and *ARID2*, respectively (Figure 5A). A total of 18,331 circRNAs and 17,453 non-differentially expressed circRNAs were detected in the Km group, accounting for 95.2%. The non-differential transcripts were subjected to target gene prediction, and 17,250 target genes were obtained. For example, Chr10: 111108887|111116249, Chr10: 34198605|34200458, Chr13: 113963909|113964098, Chr13: 23641500|23642743 targeted *OSBPL8*, *NT5DC1*, *ARL15*, and *BTN1A1*, respectively (Figure 5B).

#### 3.5.2 Functional enrichment analysis of circRNA target genes

GO and KEGG enrichment analyses were performed on the target genes of circRNAs in the Gh and Km groups, respectively. The results revealed that the most significantly enriched GO terms and KEGG pathways were associated with lactation-related processes, including Oxytocin signaling pathway, PI3K-Akt signaling pathway, JAK-STAT signaling pathway, mTOR signaling pathway, MAPK signaling pathway, prolactin signaling pathway, as well as pathways involved in glycerolipid metabolism (Figures 5C,D).

### 3.6 Identification, target gene prediction, and functional enrichment analysis of miRNAs

#### 3.6.1 Identification and target gene prediction of miRNAs

**Figure 6:**
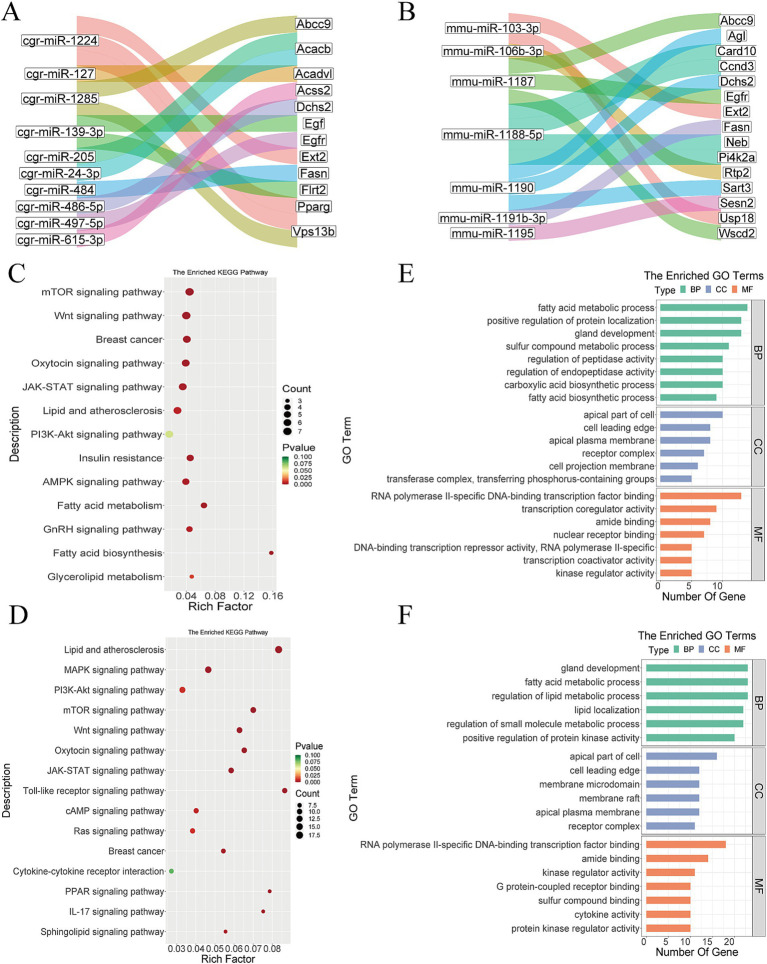
Bioinformatics analysis of miRNA. **(A)** Part of miRNA transcripts and target gene Sankey diagram in Gh group. **(B)** Part of miRNA transcripts and target gene Sankey diagram in Km group. **(C)** miRNA KEGG analysis of Gh group. **(D)** miRNA KEGG analysis of Km group. **(E)** miRNA GO enrichment analysis of Gh group. **(F)** miRNA GO enrichment analysis of Km group.

According to the data analysis, there were 1,080 miRNAs in the Gh group, of which 801 (74.2%) were non-differentially expressed. There are about 16,142 miRNAs target genes, and it was found that *VPS13B*, *EXT2*, *PPARG*, and *ACADVL* were targeted by cgr-miR-1224, cgr-miR-1224, cgr-miR-1224 and cgr-miR-127 (Figure 6A). There were 2,349 miRNAs in the Km group, of which 1,880 (80.0%) were non-differentially expressed. There were 35,035 miRNA target genes, among which mmu-miR-103-3p, mmu-miR-103-3, mmu-miR-106b-3p, and mmu-miR-1187 targeted *USP18*, *EXT2*, *RTP2*, and *EGFR*, respectively (Figure 6B).

#### 3.6.2 Functional enrichment analysis of miRNA target genes

GO and KEGG enrichment analyses were performed on the miRNA target genes of the Gh and Km groups. The results of KEGG enrichment showed that multiple pathways and biological processes were related to lactation traits, including the mTOR signaling pathway, PPAR signaling pathway, Wnt signaling pathway, oxytocin signaling pathway, JAK-STAT signaling pathway, PI3K-Akt signaling pathway, and pathways related to fatty acid biosynthesis (Figures 6C,D). In the GO analysis, pathways such as fatty acid metabolic process, regulation of lipid metabolic process, positive regulation of protein localization, and gland development were found to be closely associated with lactation (Figures 6E,F).

## 4 Discussion

The results of this study provide valuable insights into the gene expression patterns associated with lactation traits in both the Gh and Km groups. As expected, the majority of the data in both groups consisted of co-expressed genes, with a high proportion of NDEGs. This suggests that gene expression in the lactation processes of MG and MFG may be similar in both species. This observation supports our initial hypothesis that lactation-related gene expression characteristics can be effectively represented by MFG. Notably, in the circRNA data for both groups, the proportion of NDEGs approached 100%. In this study, the *CSN2*, *CSN3*, and *CSN1S1* genes, which are classified as NDEGs, exhibited high expression levels in both groups. These genes are a member of the casein gene family, mainly involved in the synthesis of milk protein, is the most important protein in mammalian milk. In addition, *LPL*, *BTN1A1*, *RPL23A*, *TUBA1C*, *ZFYVE27*, *SLC35A3*, and other NDEGs were also found, which were closely related to lactation traits. For instance, LPL has been identified as a key enzyme in lipid metabolism, playing a crucial role in determining fat composition in both adipose tissue and milk (18, 19). *BTN1A1* is one of the main proteins of milk fat globule membrane, which is related to the secretion of lipid droplets (20). Cui et al. (21) conducted transcriptional analysis on the MG of Holstein cows with great differences in milk protein and fat percentage, and found that multiple genes such as *RPL23A* can be used as candidate genes affecting milk protein and fat percentage. *TUBA1C* can be used as a potential oncogene and prognostic molecular marker in breast cancer (22). *ZFYVE27* was involved in somatic cell score. *SLC35A3* is associated with milk fat and protein percentage (23, 24). These NDEGs contribute to the stability of MG function and support lactation, playing a crucial role in the lactation process. In molecular studies investigating the underlying mechanisms of these genes, MFG can be prioritized as a non-invasive research material for studying MG-related processes, as it may serve as an effective model for understanding the role of these genes in specific lactation traits. In our data, although there are some specific expression genes and DEGs, the proportion of specific expression genes is relatively small, especially most of the genes related to lactation traits are co-expressed and non-differential.

GO and KEGG analysis showed that the two groups of NDEGs were enriched in multiple pathways, such as MAPK signaling pathway, PI3K-Akt signaling pathway, JAK-STAT signaling pathway, prolactin signaling pathway, PPAR signaling pathway, Wnt signaling pathway, fatty acid degradation, galactose metabolic and other pathways. It has been reported that MAPK and RAP1 signaling pathways can increase milk production by regulating the proliferation and differentiation of bovine MECs during the peak lactation period of dairy cows (25, 26). PPAR signaling pathway is an important lipid metabolism pathway. Changes in the expression levels of some genes in the pathway may affect the milk fat concentration in the MG (27). In addition, Lemay et al. (28) reported that the P13K-AKT pathway is highly enriched in the MG of mice during lactation. Activation of PI3K-AKT can cause breast differentiation and lead to the secretion of prolactin. In this process, the JAK-STAT pathway is also activated (29). The JAK-STAT pathway also plays a key role in regulating milk protein synthesis in non-ruminant animals (30). The above studies have confirmed the critical roles of these key pathways in lactation. In this study, we emphasize the strong correlation between the signaling pathways enriched by NDEGs and lactation traits, underscoring their potential roles in MG function and milk synthesis. Furthermore, we demonstrate that the signaling pathways enriched by NDEGs in both MFG and MG are highly similar, suggesting that the mechanisms involved in lactation in these two tissues may be conserved.

With the advancement of next-generation sequencing technology, an increasing number of ncRNAs have been identified as being associated with the lactation process. It is well established that lncRNA, circRNA, and miRNA play indispensable roles in the regulation of mammalian lactation (31–33). Related studies have examined lncRNA expression in the MG of Laoshan dairy goats at the gene expression level. The findings indicate that lncRNAs play a significant role in production, development, metabolic regulation, and fat accumulation in adipose tissue (34). Another report identified four DE-circRNAs that may regulate milk fat metabolism through circRNA screening and ceRNA network construction (35). Similarly, miRNAs play an indispensable role in important biological processes in a variety of forms. Chen et al. (36) found that chi-miR-3031 activates the PI5K-AKT-mTOR pathway by down-regulating *IGFBP3* and increases the expression of β-casein, which further provides a new experimental basis for studying the regulation mechanism of lactation and improving lactation performance. It has been reported that the inhibition of miR-199a-3p can increase the level of triglyceride and the expression of *LPL*, *ACACA*, *FABP3*, *SCD*, and *FASN* in sheep MECs, and promote the synthesis of milk fat in sheep MECs (37). These studies have collectively emphasized the regulatory potential of ncRNAs in lactation. In this study, we identified a substantial number of ncRNAs in both the Gh and Km groups, with numerous ncRNA transcripts computationally predicted to target genes related to lactation traits. These ncRNAs exert regulatory effects by modulating the expression of their target transcripts. For example, in the non-differential transcripts of the Gh group, MSTRG.3705, Chr15: 85243655|85277707, and cgr-miR-1224 were found to target *ACADVL*, *ATXN10*, and *VPS13B*, respectively. MSTRG.10612.10, Chr10:34198605|34200458 and mmu-miR-103-3p in the non-differential transcript of Km group target *EGFR*, *NT5DC1*, and *USP18* genes, respectively. It has been reported that the *ACADVL* gene has a significant genetic effect on milk yield and composition traits of dairy cows, and may be used as a genetic marker for genome selection of dairy cows (38). *VPS13B* was associated with total milk yield, fat yield and protein yield in sheep (23). Another article pointed out that *EGFR* can mediate breast development and is considered to be a key regulator of breast development (39). In addition, some scholars have carried out genome-wide association studies on lactation characteristics, milk yield and first calving age of multi-breed dairy cattle populations in Thailand. It was found that *NT5DC1* gene may be related to lactation persistence (40). Therefore, some of the ncRNA-targeted transcripts identified in this study are associated with lactation traits, suggesting that these ncRNAs may influence the lactation process through both direct and indirect mechanisms.

As previously mentioned, MFG is a potential alternative to MG samples. Building on prior research, this study systematically explored the transcriptome of both MFG and MG, further comparing and validating the feasibility of using MFG as a substitute for MG. Additionally, this study identified ncRNA targeting key lactation-related genes, expanding our understanding of how ncRNAs regulate lactation mechanisms. This is particularly significant for the more efficient use of MFG as a replacement for MG in gene function studies and molecular mechanism analysis. It is important to note that although hamsters and mice are not large dairy animals, they are commonly used in biomedical research to study lactation mechanisms due to their shared physiological processes with larger mammals and their well-defined genetic background. A major challenge in this study was RNA extraction from MFG, as it is contained in an oil-water emulsion, making it prone to degradation. Overcoming RNA degradation is crucial for obtaining reliable results. While this study provides valuable insights, further validation and comparative studies across multiple species and various non-invasive alternatives are essential to establish MFG as a widely applicable and effective substitute for MG in lactation research.

## 5 Conclusion

In conclusion, the correlation and differences in MFG and MG gene expression profiles between rodents were validated through whole transcriptome sequencing. This study provides a valuable foundation for developing non-invasive methods to study MG. However, further validation is needed to fully establish their efficacy and applicability. Although the focus was primarily on NDEGs associated with lactation, some DEGs linked to lactation traits were also identified. This study provides valuable insights into the lactation traits and related processes and offers potential support for improving dairy animal management, which may contribute to enhancing the sustainability and productivity of the dairy industry.

## Data Availability

The original sequencing data were stored in the NCBI sequence reading file (Accession No. PRJNA1003892), and located in the hamster (*Cricetulus griseus*) genome CriGri_1.0 and the mouse (*Mus musculus*) genome GRCm39.
