# Supplementary material for: Revealing gene expression links between milk fat globules and mammary glands in rodents via transcriptomics
Source: Front Vet Sci. 2025 May 13;12:1555705. doi: 10.3389/fvets.2025.1555705 (PMC12106365; doi:10.3389/fvets.2025.1555705)
Supplement: Supplementary file 1 [file Table_1.docx]

Supplementary Material

# Supplementary Tables

**Table S1.** Primers used for qRT-PCR

| **Species** | **Gene name** | **Sequence(5‘-3’)** | **Tm (℃)** | **product length**  **(bp)** | **Accession numbers** |
| --- | --- | --- | --- | --- | --- |
| Golden hamsters | *PTEN* | F:GACCATAACCCACCACAGC | 55.4 | 122 | XM_005063688.4 |
|  |  | R:CCAGTCCGTCCCTTTCCAG | 55.9 |  |  |
|  | *TLR3* | F:TGACAACCTTCCTGCTAAT | 50.0 | 168 | XM_013110874.3 |
|  |  | R:TTCAATGATGGGAGTGTTT | 50.2 |  |  |
|  | CSN3 | F:ACCTTTCTTGCCATTCCTA | 52.2 | 160 | XM_040747354.1 |
|  |  | R:CAGGGTTATTGACGGACAC | 53.0 |  |  |
|  | TUBA1C | F:GAGCGGCTCTCTGTCGATT | 56.1 | 296 | XM_040753859.1 |
|  |  | R:ATGCCAACCTTGAAGCCAGT | 56.9 |  |  |
|  | SLC35A3 | F:GACTTTGAAAGAGGAGGGT | 50.2 | 236 | XM_005077114.4 |
|  |  | R:TTGACAGTGCGACATAGAG | 48.9 |  |  |
| Kunming mice | CSN2 | F:AAAACATCCAGCCTATTGC | 52.7 | 272 | NM_001286020.1 |
|  |  | R:CTGAGAAGAAACCAGGTGAGT | 53.3 |  |  |
|  | CSN1S1 | F:ATGGCAAGTGCTCAGGAAGA | 58.4 | 220 | NM_001286015.1 |
|  |  | R:CTCTGAAGCTGTTCCAGGGT | 56.4 |  |  |
|  | CSN3 | F:CCGTGGTGAGAAGAATGAC | 52.7 | 130 | NM_001356570.1 |
|  |  | R:AGCAGTAGCAGGCAAAGAT | 52.4 |  |  |
|  | *LEP* | F:TCTTATGTTCAAGCAGTGCCTAT | 56.7 | 134 | NM_008493.3 |
|  |  | R:TCCAAGCCAGTGACCCTCT | 57.3 |  |  |
|  | *TNF* | F:CCAGGCGGTGCCTATGTCT | 60.6 | 180 | NM_001278601.1 |
|  |  | R:CCTCCACTTGGTGGTTTGTGA | 60.5 |  |  |
|  | *MAP3K5* | F:TAGGGAAGGGCACTTATGG | 50.1 | 289 | NM_008580.4 |
|  |  | R:AAGCCAATCGTCTGTTCGT | 50.2 |  |  |

**Table S2** DEGs associated with lactation traits: MG vs MFG

| Golden hamsters | | | | Kunming mice | | | |
| --- | --- | --- | --- | --- | --- | --- | --- |
| Gene | Trend | log2FC | FDR | Gene | Trend | log2FC | FDR |
| *MMP2* | down | -12.0 | 2.45×10-39 | *FLRT2* | down | -9.90 | 1.34×10-14 |
| *PALMD* | down | -8.34 | 4.50×10-15 | *ABCC9* | down | -9.76 | 3.80×10-14 |
| *FHL1* | down | -8.30 | 8.07×10-25 | *MMP2* | down | -8.74 | 1.42×10-43 |
| *ABCC9* | down | -7.64 | 3.82×10-12 | *GPIHBP1* | down | -8.71 | 1.04×10-10 |
| *BARX2* | down | -7.32 | 1.02×10-10 | *PPARG* | down | -8.67 | 1.75×10-10 |
| *OXTR* | down | -7.22 | 1.02×10-9 | *SOD3* | down | -8.65 | 2.89×10-22 |
| *CTNND2* | down | -6.55 | 3.75×10-8 | *CCL2* | down | -8.43 | 1.65×10-9 |
| *COCH* | down | -6.40 | 8.11×10-8 | *CHP2* | down | -8.01 | 7.81×10-9 |
| *WSCD2* | down | -6.12 | 3.60×10-7 | *FHL1* | down | -7.18 | 8.07×10-17 |
| *CHP2* | down | -5.15 | 3.92×10-8 | *FAT4* | down | -7.16 | 2.37×10-31 |
| *GOLGA7B* | up | 9.21 | 1.66×10-16 | *ATP2B2* | up | 6.17 | 1.86×10-43 |
| *EGF* | up | 6.59 | 1.77×10-13 | *EGF* | up | 4.73 | 1.58×10-22 |
| *ATP2B2* | up | 6.24 | 2.50×10-9 | *C2CD4B* | up | 4.09 | 2.53×10-9 |
| *LPL* | up | 6.21 | 1.15×10-16 | *ACACA* | up | 4.06 | 4.4×10-19 |
| *FABP3* | up | 5.65 | 1.05×10-10 | *FASN* | up | 4.05 | 3.55×10-13 |
| *LALBA* | up | 5.34 | 4.83×10-10 | *FABP3* | up | 4.04 | 8.62×10-10 |
| *CEL* | up | 4.90 | 6.57×10-9 | *ACLY* | up | 3.44 | 5.17×10-13 |
| *FASN* | up | 4.58 | 2.43×10-11 | *PTHLH* | up | 3.41 | 2.89×10-5 |
| *CSN2* | up | 4.51 | 4.01×10-7 | *ACACB* | up | 2.92 | 1.29×10-9 |
| *KCNK1* | up | 3.36 | 1.40×10-7 | *ESRRG* | up | 2.84 | 1.73×10-10 |

**Table S3** NDEGs associated with lactation traits

| Golden hamsters | | | | Kunming mice | | | |
| --- | --- | --- | --- | --- | --- | --- | --- |
| Gene | Trend | log2FC | FDR | Gene | Trend | log2FC | FDR |
| *CSN3* | normal | 2.01 | 0.11 | *CSN2* | normal | 0.72 | 0.34 |
| *TUBA1C* | normal | 0.33 | 0.22 | *CSN1S1* | normal | 1.3 | 0.09 |
| *RPL23A* | normal | 0.91 | 0.02 | *CSN3* | normal | 0.4 | 0.45 |
| *PDIA3* | normal | 0.6 | 0.2 | *GLYCAM1* | normal | 1.54 | 0.13 |
| *RPL27A* | normal | 0.37 | 0.82 | *MFGE8* | normal | -0.96 | 0.03 |
| *PTEN* | normal | -0.22 | 0.34 | *CD14* | normal | -0.59 | 0.54 |
| *CD74* | normal | -0.4 | 0.8 | *BTN1A1* | normal | 0.33 | 0.42 |
| *GYG1* | normal | 0.63 | 0.24 | *CEL* | normal | 0.96 | 0.23 |
| *ALOX5AP* | normal | -1.25 | 0.36 | *XDH* | normal | 0.96 | 0.04 |
| *WIPI1* | normal | 0.53 | 0.18 | *MUC1* | normal | -0.43 | 0.34 |
| *ID2* | normal | -0.54 | 0.35 | *ARF1* | normal | 0.55 | 0.06 |
| *GRINA* | normal | 0.27 | 0.53 | *LPL* | normal | -0.1 | 0.87 |
| *RORA* | normal | 0.93 | 0.13 | *SLC7A5* | normal | -0.41 | 0.39 |
| *EIF4E* | normal | 0.46 | 0.17 | *GPX3* | normal | 0.12 | 0.89 |
| *NFKB1* | normal | -0.35 | 0.57 | *LDB1* | normal | 0.2 | 0.58 |
| *GNG10* | normal | 0.95 | 0.09 | *PRDX5* | normal | 0.11 | 0.8 |
| *MFSD14A* | normal | 0.51 | 0.13 | *ETS2* | normal | -0.67 | 0.14 |
| *SMARCA5* | normal | -0.46 | 0.09 | *TUBA1C* | normal | -0.27 | 0.61 |
| *AGAP3* | normal | -0.31 | 0.67 | *CDKN1A* | normal | -1.23 | 0.11 |
| *PPARG* | normal | -0.15 | 0.94 | *GOT1* | normal | 0.93 | 0.01 |
